# How to translate therapeutic recommendations in clinical practice guidelines into rules for critiquing physician prescriptions? Methods and application to five guidelines

**DOI:** 10.1186/1472-6947-10-31

**Published:** 2010-05-28

**Authors:** Jean-Baptiste Lamy, Vahid Ebrahiminia, Christine Riou, Brigitte Seroussi, Jacques Bouaud, Christian Simon, Stéphane Dubois, Antoine Butti, Gérard Simon, Madeleine Favre, Hector Falcoff, Alain Venot

**Affiliations:** 1 Laboratoire d'Informatique Médicale et de Bioinformatique (LIM&BIO), UFR SMBH, University of Paris 13, 74 rue Marcel Cachin, 93017 Bobigny cedex, France; 2 Université Paris 12, UFR de Médecine, Créteil, France; AP-HP, Hôpital Henri Mondor, Département d'Information Hospitalier, Créteil, France; 3 U936 INSERM, University of Rennes 1, IFR 140, Rennes, France; 4 Université Paris 6, UFR de Médecine, Paris, France; AP-HP, Hôpital Tenon, Département de Santé Publique, Paris, France; 5 AP-HP, DSI, STIM, Paris, France; INSERM, UMR_S 872, eq. 20, Paris, France; 6 Silk Informatique, 40 bis avenue du général Patton, 49000 Angers, France; 7 RESIP, Centre Directionnel 56 rue Ferdinand Buisson BP 455, 62206 Boulogne sur Mer Cedex, France; 8 ESIRIM, 39 allée de la Pitancerie F94230 Cachan, France; 9 Université Paris Descartes, Faculté de Médecine, Département de Médecine Générale, 75015 Paris; Société de Formation Thérapeutique du Généraliste (SFTG), 233 bis rue de Tolbiac, 75013 Paris, France

## Abstract

**Background:**

Clinical practice guidelines give recommendations about what to do in various medical situations, including therapeutical recommendations for drug prescription. An effective way to computerize these recommendations is to design critiquing decision support systems, *i.e*. systems that criticize the physician's prescription when it does not conform to the guidelines. These systems are commonly based on a list of "if conditions then criticism" rules. However, writing these rules from the guidelines is not a trivial task. The objective of this article is to propose methods that (1) simplify the implementation of guidelines' therapeutical recommendations in critiquing systems by automatically translating structured therapeutical recommendations into a list of "if conditions then criticize" rules, and (2) can generate an appropriate textual label to explain to the physician why his/her prescription is not recommended.

**Methods:**

We worked on the therapeutic recommendations in five clinical practice guidelines concerning chronic diseases related to the management of cardiovascular risk. We evaluated the system using a test base of more than 2000 cases.

**Results:**

Algorithms for automatically translating therapeutical recommendations into "if conditions then criticize" rules are presented. Eight generic recommendations are also proposed; they are guideline-independent, and can be used as default behaviour for handling various situations that are usually implicit in the guidelines, such as decreasing the dose of a poorly tolerated drug. Finally, we provide models and methods for generating a human-readable textual critique. The system was successfully evaluated on the test base.

**Conclusion:**

We show that it is possible to criticize physicians' prescriptions starting from a structured clinical guideline, and to provide clear explanations. We are now planning a randomized clinical trial to evaluate the impact of the system on practices.

## Background

Clinical practice guidelines (CPGs) provide recommendations for the diagnosis and treatment of numerous diseases; they have been proved to be helpful for physicians [1]. However, guidelines printed on paper are difficult to use efficiently during medical consultation [2] and guideline-based learning programmes are not sufficient [3]. This has led to the development of decision support systems (DSSs) based on CPGs. Two reviews reveal that DSSs improved clinical practices in 64% [4] and 68% [5] of trials, and the use of a DSS was identified as one of the factors critical for success in improving healthcare for chronic disease [6,7]. In particular, *critiquing DSSs*, requiring little or no intervention from the physician, provide criticism to the physician whenever his/her activity (*e.g*. drug prescriptions) is considered by the DSS as non-adequate in the light of current medical knowledge [8]. Critiquing DSSs have been shown to have a greater impact than on-demand DSSs on practice [4,9].

A first approach for designing critiquing DSSs consists in modelling the situations and actions that should be criticized, typically using a set of "if conditions then criticism" rules. It has been shown that if-then rules are satisfactory for critiquing drug prescriptions on the basis of the therapeutical recommendations expressed in many CPGs [10]. Critiquing DSSs based on "if conditions then criticism" rules have been proposed for various medical problems, including asthma [11,13], dyslipaemia [9,13], antibiotic prescriptions [13], and test ordering [14,15]. However, building the knowledge base requires converting CPG recommendations into these "if conditions then criticism" rules. This task is difficult because:

1. it requires both logical and medical expertise, and therefore it needs input from both physicians and computer scientists,

2. it requires to take into account medical knowledge that is implicit in the CPGs *(e.g*. CPGs do not explicitly state that it is possible to reduce the dose of a drug to lower the adverse effects it causes),

3. there is not one-to-one mapping between recommendations and criticisms; for instance the following recommendation "at the first stage of diabetes type 2, prescribe metformin as first-line treatment, and an alpha-glucosidase inhibitor (AGI) as second-line treatment" leads to three possible criticisms (see table 1):

**Table 1:** Various possible situations for an example of therapeutical recommendation.

	physician proposed metformin (first-line treatment)	physician proposed alpha-glucosidase inhibitors (AGI, second-line treatment)	physician proposed any other treatment
patient at the stage of first-line treatment	OK	criticism: AGI should be prescribed only as second-line treatment. Guideline recommends metformin as first-line treatment.	criticism: Sulfonamides, glinides and glitazones are not recommended. Guideline recommends metformin as first-line treatment.

patient at the stage of second-line treatment	OK (*e.g*. with a different dose; preventing the represcription of ineffective or poorly tolerated treatments is the task of other recommendations)	OK	criticism: Sulfonamides, glinides and glitazones are not recommended. Guideline recommends metformin as first-line treatment, and AGI as second-line.

The table shows the six possible cases for a given patient and physician prescription with regard to the recommendation "prescribe metformin as first-line treatment and alpha-glucosidase inhibitor (AGI) as second-line".

(a) if AGI is prescribed as first-line treatment: "AGI is a second-line treatment; metformin is recommended as first-line treatment",

(b) if another drug is prescribed as first-line treatment: "other drugs are not recommended for the patient; metformin is recommended as first-line treatment",

(c) if another drug is prescribed as second-line treatment: "other drugs are not recommended for the patient; metformin is recommended as first-line treatment and AGI as second-line".

Despite the two first criticisms lead to the same recommendation (prescribe metformin), the criticism displayed to the physician should not be the same, since the reasons and the explanations justifying the alert are different.

In ASTI 1 and ASTI 2 [16,18], we proposed another approach for establishing critiquing DSSs that uses a structured model of the CPG therapeutical recommendations. The system first determines the set of drug prescriptions recommended for the patient, and then raises an alert if the physician's prescriptions are not in this set. During preliminary tests, this approach efficiently detected physician's prescriptions that did not conform to the CPG [19]. However, it failed to generate a textual critique explaining to the physician why the drugs he/she prescribed did not conform, because (1) the first part of the reasoning process (determining the drugs recommended for the patient) does not take into account the physician's prescription, and (2) the knowledge model was limited to the representation of recommendations, which was insufficient to generate a meaningful critique (see difficulties 2 and 3 above). For instance, when applying the recommendation of the previous example to the prescription of an AGI for a patient as first-line treatment, the system deduces that the only recommended prescription is metformin. As the physician's prescription is different, a critique is generated. However, the textual critique is limited to "metformin is recommended as first-line treatment", without being able to state that "AGI is a second-line treatment" as above.

The objective of this article is to present and evaluate a drug prescription critiquing system that combines the two approaches presented above, and aims at (1) facilitating the creation of new knowledge bases, with a system designed to support the therapeutical recommendations of many, if not all, CPGs, and (2) being able to generate a clear and appropriate textual critique that explains to the physician *why *his/her prescriptions do not conform to the CPG. The knowledge base is composed of (a) specific recommendations that directly match the CPG recommendations, enriched with textual labels for generating the critiques, and (b) generic recommendations that model the implicit, CPG-independent, medical knowledge required for the critiquing process. This knowledge base is then automatically transformed into a set of executable "if conditions then criticism" rules. To show that it is easy to write knowledge bases and that the system is generic, we applied it to the therapeutic recommendations of five CPGs concerning chronic diseases related to cardiovascular risk.

The article first presents the ASTI project, as part of which this study was carried out. Then we present the methods for selecting the CPGs, designing the models, writing the algorithms, and evaluating the system. The results section describes the system by presenting the models, the algorithms for translating recommendations into "if conditions then criticism" rules, and the generic recommendations, and gives the results of the implementation of the five CPGs and the evaluation for these CPGs. Finally, we consider the value and the limitations of the DSS we have developed.

## The ASTI project

**Figure 1:**
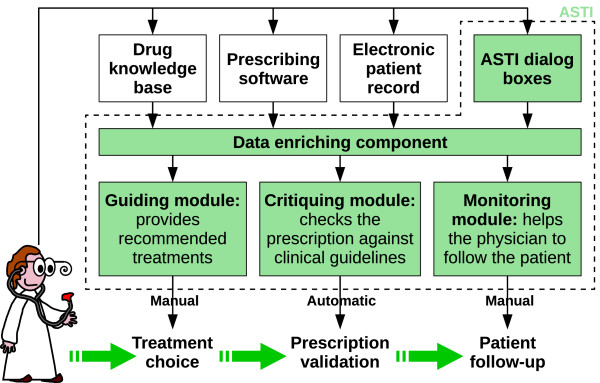
**Global architecture and components of the ASTI project**.

The ASTI project [16,18] aims to improve therapeutic care for patients with chronic diseases, through the design of generic DSSs which may help physicians to take into account the therapeutic recommendations of relevant CPGs. The global architecture of the ASTI project is shown in figure 1. The project includes three complementary modules that correspond to various steps in medical care [20]. The first module is the guiding module which provides the physician with recommended treatments through an hypertextual navigation within the knowledge base [21]. The second module, the critiquing module, is for validating prescriptions. It is automatically activated when the physician writes a prescription; it generates an alert when the prescription does not conform to the CPG. This module was inspired by the reminder systems successfully developed over recent decades for preventing drug interactions. The third module concerns patient follow-up; it lists various clinical and biological variables for the patient, with additional temporal data, such as when the physician should prescribe biological tests, according to the CPG recommendations. This article focuses on the critiquing module. The ASTI project has evolved over the last few years: ASTI 1 and 2 were initially aimed at hypertension and diabetes type 2, and ASTI 3 now aims to generalize the progress made to other CPGs.

## Methods

### Design methods

We started with the ASTI 2 critiquing module, which provided a general architecture for the DSS. The ASTI 2 critiquing module is a rule-based system, with (a) a data-enriching component, which computes derived patient data from data available in the patient files, *e.g*. computing the Body Mass Index (BMI) from the height and the weight, (b) an inference engine, and (c) two knowledge bases implementing the French CPGs for hypertension [22] and type 2 diabetes [23].

#### Selecting guidelines

We worked on five CPGs published by the French health authorities for hypertension [22], type 2 diabetes [23], tobacco addiction [24], dyslipaemia [25] and atrial fibrillation [26]. These CPGs were chosen because they all relate to the cardiovascular risk and cover various aspects of the clinical care, *e.g*. the tobacco addiction CPG involves short-duration treatments whereas the other CPGs involve life-long treatments.

#### Modelling "if conditions then criticism" rules

*"If conditions then criticism" rules *are executed by the inference engine. Various elements were considered for rule conditions, inspired by the ASTI 2 critiquing module: (a) the patient's clinical condition (including current and past diseases and physiological states), (b) biological test results for the patient, and (c) the patient's therapeutic history (*i.e*. the list of past and current prescriptions), including treatment outcomes *(i.e*. treatment efficacies and drug tolerances). In the CPGs, clinical and biological conditions are relatively simple. Treatments expressed in CPGs are more complex, because many levels of granularity are used. We started from the ASTI 2 treatment model [18], and we extended this model to represent not only precise treatments, but also what we call hereafter *treatment patterns *of a lower granularity, such as "any bitherapy", "metformin in bitherapy" or "any past treatment including metformin with poor tolerance".

#### Modelling CPG recommendations

In the ASTI 3 critiquing module knowledge base, *CPG recommendations *are written manually from the guidelines; they aim at being easy to write and as close as possible to the CPG.

We extracted all therapeutic recommendations from the five CPGs, and for structuring them we designed a simple model inspired by the plan-based models in the literature. Each recommendation can lead to one or more criticisms. For instance, if we consider the recommendation "prescribe metformin as first-line treatment and alpha-glucosidase inhibitor (AGI) as second-line", there are two patient stages (patient requiring first-line treatment and patient requiring second-line treatment) and three treatments the physician can prescribe (metformin, AGI, and any other), and thus six possible situations, shown in table 1. Three of them lead to a criticism, all three criticisms being different. Therefore, we enriched the CPG recommendation model with attributes for modelling the textual criticisms.

Many guideline recommendations are complex and involve several lines of treatment. Recommendations with several lines of treatments could theorically be split in simpler recommendations, though it is not always desirable; for instance "prescribe metformin as first-line treatment" and "prescribe AGI as second-line treatment". However the second of these recommendations cannot be interpreted alone: "second-line treatment" is actually relative to the first recommendation and actually means "if metformin cannot be prescribed or was not satisfying". Therefore, we didn't try to split recommendations.

#### Writing generic recommendations

Pieces of medical knowledge that are both well-known by physicians and not specific to the disease addressed by the CPG are usually implicit in guidelines. For example, CPGs do not explicitly state that it is possible to reduce the dose of a drug to lower the adverse effects it causes. However, such medical knowledge is necessary for critiquing a prescription.

Consequently, we wrote *generic recommendations *for capturing this implicit knowledge. The criteria for a generic recommendation are the following: (a) it is independent of CPG, (b) it applies to at least three of the knowledge bases we developed for the five CPGs listed above, and (c) it is likely to apply to other CPGs. We required at least three occurrences (criterium b), because many recommendations are guideline-specific, even if they do not involve drugs or clinical contexts related to the guideline's disease, *e.g*. when a monotherapy has no effect at all, the guideline for arterial hypertension recommends to try another drug, but not to prescribe a bitherapy; however this recommendation is not found in the other guidelines, and therefore it cannot be considered as generic. By default, generic recommendations apply to all knowledge bases; for some of them it is possible to specify exceptions, *e.g*. lowering the dose of the bupropion is not possible, due to its narrow therapeutic range.

#### Designing algorithms for transforming recommendations into "if conditions then criticism" rules

The final step in building the ASTI 3 critiquing module was to design algorithms for automatically transforming both CPG and generic recommendations into "if conditions then criticism" rules. First, we did a preliminary feasibility study to ensure that such translation was possible. In this study, a set of "if conditions then criticism" rules equivalent to the recommendations for antihypertensive monotherapy were written manually. We chose antihypertensive monotherapy because it contains substantial complexity in a small subset of a CPG. Then, we wrote algorithms for automating the translation of recommendations into "if conditions then criticism" rules. One of the most complex types of recommendations follows the pattern "prescribe X as first-line treatment, Y as second-line treatment,..."; an example of such recommendations is given in table 1. We generalized this situation to N line of treatments.

Some of the generic recommendations are translated into "if conditions then criticism" rules that are totally independent from the content of the CPG (*e.g*. a rule critiquing the interruption of an effective and well-tolerated treatment). Some other generic recommendations lead to algorithms that generate one "if conditions then criticism" rule for each pharmaco-therapeutic class of drug or for each treatment recommended by the CPG (*e.g*. a rule critiquing the represcription of metformin if metformin has been poorly tolerated in the past). Finally, the remaining generic recommendations were taken into account when writing the various algorithms, but were not used to produce "if conditions then criticism" rules directly.

The complete list of algorithms is reported in the results section.

#### Software implementation methods

The ASTI 3 critiquing module was written using the Python programming language. The therapeutic history was coded using the ATC (Anatomical Therapeutic Chemical) drug classification.

### Testing and evaluation methods

Three types of tests were performed to ensure the conformity of the DSS recommendations to the CPGs content. In all tests, we considered the guidelines as the "gold-standard", and thus we didn't investigate potential error in the guidelines.

First, a test base was written manually for each CPG. The test base was built by creating patient profiles that covered the various clinical situations and types of treatment. Then for each patient profile, we generated several test cases corresponding to the prescription of various treatments to that patient: one test case per treatments recommended by the CPG, and five test cases with randomly-generated non-recommended treatments, in order to verify that these non-recommended treatments are criticized as expected.

Second, for the diabetes type 2, tobacco addiction, dyslipaemia, atrial fibrillation and thrombo-embolic risk knowledge bases, a new quasi-exhaustive verification method was used [19]; this method considers the DSS as a black box, and tries to regenerate the CPG knowledge from the DSS. It consists in three steps: (1) Generating an almost exhaustive set of the possible DSS input vectors. This was achieved by considering a limited number of patient attributes *(e.g*. age, sex, current treatment,...) and a limited number of possible values for each attribute *(e.g*. 14, 18, 35, 75 years for age), and then generating all the possible combinations of attribute values. Finally, the DSS was run to determine the output for each input vector. (2) Extracting knowledge from the set of (input vector, output result) pairs. We used the C4.5 algorithm to generate a decision tree; pruning was disabled to keep 0% of error in the tree. (3) Comparing the decision tree generated with the original CPG, to check that the treatments recommended by the tree conform to the CPG, and that none of the recommendations included in the CPG are missing from the tree. This method was not applied to the hypertension knowledge base, because the number of possible input vectors was too high, and the decision tree would be far too big to be human-readable. Third, the knowledge bases were reviewed manually by a physician, who was asked to compare them to the content of the original CPGs. The physician was briefly introduced to the functioning of the ASTI 3 critiquing module. The recommendations in the knowledge bases were rephrased into an equivalent text in natural language, before being reviewed by the physician, *e.g.: *"if the treatment prescribed is a monotherapy and HbA1c ≤ 6.5%, then metformin should be prescribed as first-line treatment, and AGI as second-line treatment".

## Results

### Description of the ASTI 3 critiquing module

**Figure 2:**
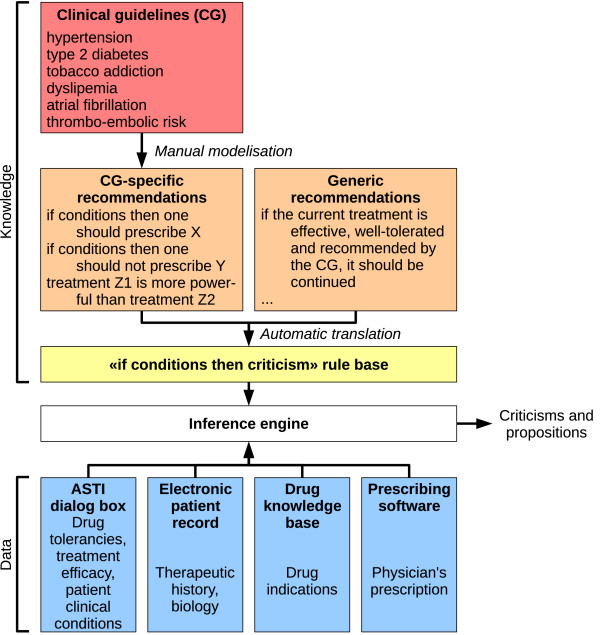
**Architecture and data sources of the ASTI critiquing module**.

The general architecture of the ASTI critiquing module is shown in figure 2. The ASTI critiquing module was linked to éO Généraliste^®^, a French Electronic Patient Record (EPR) which provides the therapeutic history, the biological results and some clinical conditions of the patient. Additional dialog boxes were added to éO Généraliste^® ^to ask for treatment outcomes and further information about patient clinical condition. The ASTI 3 data enriching component is responsible for linking the critiquing module to the EPR. In particular, the data enriching component determines the indication of each prescribed drug, from the drug's recommended indication and the prescribed dose (*e.g*. for aspirin, which have different indication depending on the dose), and regroups the drugs by indication, possibly duplicating them when a drug has several indications *(e.g*. beta-blocking agents are indicated for both arterial hypertension and atrial fibrillation). Finally, the critiquing module is run for each indication found in the prescription.

The data enriching component is also in charge of computing various medical abstractions, such as relative posologies (i.e. has the drug dose been increased or lowered in the new prescription?). A few elements are required for critiquing the prescription, but are usually not present, or only in free text, in the EPR: drug tolerancies, treatment efficacy, and some clinical conditions (*e.g*. late discovery of diabetes type 2). For these elements, dialog boxes have been added to the EPR, for asking them to the physician. The values of these elements are then stored in the EPR for future uses.

In the following subsections, we describe the various parts of the critiquing module.

#### "If conditions then criticism" rule model and treatment pattern model

As stated in the introduction, "if conditions then criticism" rules are not supposed to be manually written, but automatically generated from a model of the CPG. Rule conditions can include clinical elements, represented by simple (attribute, operator, value) triplets (*e.g*. (age, inferior to, 75) or (diabetic, equal, yes)), and therapeutic elements, represented by treatment patterns. AND, OR and NOT logical operators can be used to combine several elements in conditions, and these operators can be nested.

**Figure 3:**
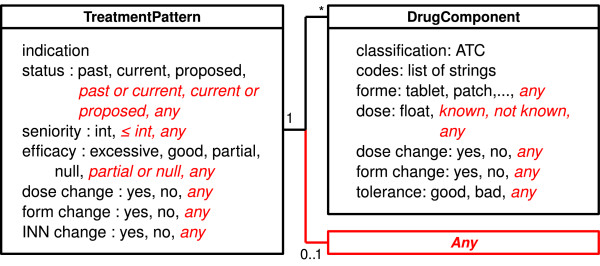
**The treatment pattern model, represented in UML**. The elements that were added to extend the treatment model into a treatment pattern model are shown in red and italics. INN means "International Nonproprietary Name".

Treatment patterns are queries that can be read as: "it exists in the therapeutic history at least one treatment that matches the pattern X". The treatment pattern model is shown in figure 3; this model can represent treatment of various levels of granularity, from a specific treatment to classes of treatment (*e.g*. bitherapy, insulinotherapy) and partially defined treatments (*e.g*. any treatment including metformin). It contains information about (a) drug classes (using the ATC code; the model allows to define a drug class using several codes because some pharmacological classes cannot be represented by a single ATC code, *e.g*. glinide), (b) dose and form, (c) changes from the preceding treatment (dose change, form change, INN change), (d) drug tolerance and treatment efficacy for the patient (these items of information are specified by the physician in ASTI 3 dialog boxes), (e) treatment status (past treatment, current treatment or treatment proposed by the physician and being validated by the critiquing module).

The criticism part of the rules is simply represented by a textual label to be presented to the physician.

#### CPG recommendation model

**Figure 4:**
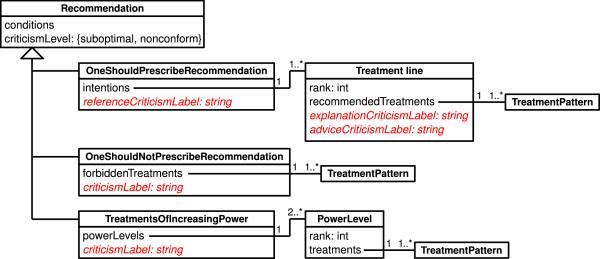
**The recommendation model, represented in UML**. Attributes modelling the textual criticism are shown in red and italics.

We structured the therapeutical recommendations found in CPGs. Our work with five CPGs led us to distinguish three types of recommendations: (1) *"one should prescribe" *recommendations give a list of recommended treatments. There can be several lists corresponding to first, second,... N^th ^lines of treatments, and we associate line N + 1 to any other non-recommended treatment (the patient is never considered to be at the stage of treatment of line N + 1, but the treatment prescribed by the physician can be a treatment of line N + 1, if it does not match any other line), (2) *"one should not prescribe" *recommendations advise to not prescribe some treatments in a given situation, and (3) *"treatments of increasing power" *recommendations state that some treatments are more effective than others. The recommendation model is presented in figure 4; conditions and treatment patterns are the same as those used in "if conditions then criticism" rules.

In this model, "criticism label" attributes are used for modelling the textual criticisms shown to the physician, usually using excerpts of the CPG. For "one should prescribe" recommendations, the criticism label is split into three parts: (1) the explanation criticism label explains why a treatment of a given line should not be prescribed to a patient requiring a treatment of a lower line *(e.g*. a second-line treatment to a patient at the stage of first-line treatment), (2) the advice criticism label states the recommended treatments for patients require a treatment of a given line, and (3) the reference criticism label gives bibliographic references *(e.g*. the page number in the CPG). There are one advice and explanation criticism labels for each line of treatment in the recommendation. The criticism shown to the physician will be the concatenation of the explanation criticism label of the line of the treatment proposed by the physician, the advice criticism label of the line at which the patient is, and the reference criticism label.

In the example in table 1, there are 2 lines of treatment in the recommendation, thus line 3 is the "any other treatment" line (the last column). In the three criticisms, the first sentence is the explanation part and the second the advice part (the reference part is not shown). As a result of the model, advice criticism labels are the same in each line, and explanation criticism labels are the same in each column.

#### Generic recommendations

Eight generic recommendations were found during the design of the ASTI 3 critiquing module; they are listed in table 2. These recommendations correspond to a sort of "medical common sense"; they are related to the treatment and apply to many, and in some case all, medical situations. They are either independent from CPG, or they depend only on the list of pharmacological drug classes or treatments that the CPG recommends. Generic recommendation #1 leads to continuing an effective well-tolerated treatment: this is logical for chronic diseases. Generic recommendations #2 to #4 are related to dose; they provide generic guidance but can be overridden by CPG-specific rules, *e.g*. tolerance of some drugs is not dose-dependent. Generic recommendations #5 and #6 prevent represcription of a treatment that has failed in the past; we limited generic recommendation #6 to the recent past because a treatment that was ineffective years ago may be effective in the future if the patient's conditions are different *(e.g*. if the patient's weight has changed substantially). Generic recommendation #7 leads to apply the recommendations for poor tolerance when the treatment is both ineffective and poorly tolerated, because in case of poor tolerance, the patient's adherence to the treatment is often low and therefore the efficacy of the treatment cannot be rigorously evaluated. Finally, generic recommendation #8 prevents the simultaneous prescription of two drugs of the same pharmacological class (*e.g*. two beta-blocking agents).

**Table 2:** The generic recommendations and their use in the knowledge bases.

#	Generic recommendations	**Hyp**.	**Diab**.	**Dys**.	**Tob**.	**Atr**.	**Thr**.	Exceptions
1	If the current treatment is effective, well-tolerated and recommended by the CPG, it should be continued	●	●	●	○	●	○	12/113

2	If the current treatment is ineffective, the dose can be increased	◐	●	●	◐	●	●	2/113

3	If the current treatment is too effective, the dose can be decreased	●	●	n.a.	n.a.	n.a.	●	0/113

4	If a drug of the current treatment is poorly tolerated, the dose can be decreased	●	●	●	◐	●	●	1/43

5	If a treatment was not effective in the recent past, it should not be prescribed again	●	●	●	○	●	○	15/113

6	If a drug was not tolerated in the past, it should not be prescribed again	●	●	●	●	●	●	0/43

7	If a treatment is both poorly tolerated and ineffective, apply the recommendations for poor tolerance	●	●	●	●	●	●	0/113

8	Two drugs of the same pharmaco-therapeutic class should not be prescribed in association	●	◐	●	◐	●	●	6/43

Hyp.: hypertension, Diab.: type 2 diabetes, Dys.: dyslipaemia, Tob.: tobacco addiction, Atr.: atrial fibrillation, Thr.: thrombo-embolic risk. ● indicates that the recommendation is used for implementing the CPG, ◐ that the recommendation is used with exceptions, ○ that the recommendation does not apply, and n.a. that the situation does not occur in practice. The exception column gives the rate of exceptions for each generic recommendations (number of exceptions/denominator; the denominator is either 43, the total number of drug classes, or 113, the total number of recommended treatments found in the five guidelines).

#### Algorithms for transforming recommendations into "if conditions then criticism" rules

We wrote algorithms for transforming CPG and generic recommendations into "if conditions then criticism" rules. The conditions of the rules generated include the conditions from the recommendations (labelled "(condition)" below) and additional conditions.

-For a "one should not prescribe" recommendation, the algorithm is trivial and generates one rule: "if (conditions) and (the treatment proposed by the physician is the treatment to not prescribe) then criticism".

-For a "treatment of increasing power" recommendation with N power levels, the algorithm generates N - 1 rules: "if (conditions) and (the proposed treatment is a treatment of power level X) and (there is in the therapeutic history an ineffective treatment of power level Y > X) then criticism" (for 2 ≤ X ≤ N).

-For a "one should prescribe" recommendation with N lines of treatments (line N + 1 being associated to any other treatment), the algorithm generates  rules. We consider that the patient is at the stage of line X if and only if:

*when X = 1, "there is no ineffective or poorly tolerated treatment of any line in the therapeutic history",

*when 2 ≤ X ≤ N - 1, "(there is no ineffective or poorly tolerated treatment of line ≥ X in the therapeutic history) and (there is an ineffective or poorly tolerated treatment of line X - 1 in the therapeutic history)",

*when X = N, "there is an ineffective or poorly tolerated treatment of line N - 1 or N in the therapeutic history".

The rules are: "if (conditions) and (the patient is at the stage of line X) and (the proposed treatment is a treatment of line Y) and (the proposed treatment is not a treatment of line < Y) then criticism (explanation criticism label for line Y + advice criticism label for line X + reference criticism label)" (for 1 ≤ X ≤ N, for X + 1 ≤ Y ≤ N + 1).

We include "the proposed treatment is not a treatment of line < Y" in conditions because, due to the various possible levels of granularity for expressing treatments, there might be overlap between treatments from different lines. *E.g*. in the recommendation "prescribe simvastatin or pravastatin as first-line treatment, and any statin as second-line treatment".

-Generic recommendation #1 leads to the rule: "if (the current treatment conforms to the guideline, and is effective and well-tolerated) and (the proposed treatment includes an INN change, a dose change or a form change) then criticism".

-Dose-related generic recommendations (#2, 3 and 4) lead to three rules:

*"if (the current treatment is ineffective but well-tolerated) and (the proposed treatment is a dose reduction) then criticism"

*"if (the current treatment is poorly tolerated) and (the proposed treatment is a dose increase) then criticism"

*"if (the current treatment is too effective) and (the proposed treatment is a dose increase) then criticism". An example of a too-effective treatment is an antivitamin K anticoagulant drug, when the INR (International Normalized ratio) is higher than the therapeutical range, i.e. the anticoagulant effect is too important, and the drug dose should be reduced.

-For generic recommendation #5, we consider that a past ineffective treatment should not be represcribed if it has been stopped in the past. This leads to one rule for each recommendable treatment T in the guideline: "if (the proposed treatment is a treatment T) and (the therapeutic history includes an ineffective treatment T within the last three years, which has not been followed by another treatment T) then criticism".

-Similarly, for generic recommendation #6, we consider that a drug poorly tolerated in the past should not be represcribed if it has been stopped in the past. This leads to one rule for each pharmaco-therapeutic class C in the guideline: "if (the proposed treatment includes a drug of the pharmaco-therapeutic class C) and (the therapeutic history contains a past treatment including a poorly tolerated drug of the pharmaco-therapeutic class C, which was followed by a treatment that does not include a drug of the pharmaco-therapeutic class C) then criticism".

-Generic recommendation #8 leads to one rule for each pharmaco-therapeutic class C in the guideline: "if (the proposed treatment includes two drugs of the pharmaco-therapeutic class C) then criticism".

#### Description of the knowledge bases

It was possible to represent all therapeutic recommendations found in the five CPGs using the CPG recommendation model. The CPG for atrial fibrillation included recommendations for two clinical conditions: atrial fibrillation itself and thrombo-embolic risk; consequently, we wrote a separate knowledge base for each. For both of them, the CPG takes into account some clinical variables that evolve very frequently (*e.g*. patient at fibrillation for less than 48 hours) or that might not be available at the time of prescription (*e.g*. the type of atrial fibrillation, either paroxistic, permanent or persistent, is frequently known a *posteriori*, since it depends on the presence of recurrences in the next 7 days). Consequently, we wrote partial knowledge bases that do not take these variables into account. Table 3 shows the characteristics of the six knowledge bases, and the generic recommendations which apply in each knowledge base are given in table 2. Additional file 1 gives examples of recommendations in ASTI 3, and Additional file 2 shows examples of generated rules.

**Table 3:** Characteristics of the knowledge bases.

		**Hyp**.	**Diab**.	**Dys**.	**Tob**.	**Atr**.	**Thr**.
Modelled	Number of pharmaco-therapeutic drug classes	7	7	10	5	11	3
	
CPG	Number of recommended treatments	30	39	15	12	14	3
	
recommendations	Number of "one should prescribe..." recommendations	30	14	9	7	6	6
	
	Number of "one should not prescribe..." recommendations	19	10	6	11	4	1
	
	Number of "treatments of increasing power..." recommendations	1	3	2	1	0	0
	
	Total number of recommendations	50	27	17	19	10	7

Generated rules	Number of "if ... then criticism" rules	119	102	73	42	51	16
	
	Number of treatment patterns	826	1121	708	267	591	82

Hyp.: hypertension, Diab.: type 2 diabetes, Dys.: dyslipaemia, Tob.: tobacco addiction, Atr.: atrial fibrillation, Thr.: thrombo-embolic risk. User-defined knowledge bases refer to the recommendations as entered into the system, and generated knowledge bases to the rule bases automatically generated by the system, including generic rules and after applying macros to transform all recommendations into "if conditions then criticism" rules.

#### Inference engine

The inference engine is simple, most of the reasoning being done by the algorithms described above. It performs the following four steps:

(1) it executes the "if condition then criticism" rules for the treatment proposed by the physician; if one or more rules is triggered, the treatment does not conform to the knowledge base and an alert will be issued,

(2) it generates a textual criticism by concatenating the textual criticisms of all triggered rules,

(3) it generates a list of treatment suggestions, by executing the "if condition then criticism" rules for all recommendable treatments in the CPG, and retaining only the treatments that do not trigger any rule,

(4) if the list of suggestions is empty, the rules are relaxed to accept a second-line treatment for a patient at the stage of first-line treatment (or a third-line treatment for a patient at the stage of second-line treatment, etc), and the inference engine restarts at step 1. If the list of suggestions is still empty, the rules can again be relaxed, to accept a third-line treatment for patient at the stage of first-line treatment, and so on. This situation occurs when a line of treatment cannot be prescribed, *e.g*. because all the recommended drugs are not tolerated by the patient; in that case, the usual prescribing behaviour is to prescribe a second-line (or third-line, etc) treatment. If the list of suggestions is empty when relaxing the rules to the maximum, then the CPG does not provide enough information for making a decision *(e.g*. all the possible treatments are contraindicated, poorly tolerated or ineffective).

### Testing and evaluation results

We first tested the ASTI critiquing module using a test base involving 59 clinical cases and 652 test cases for hypertension, 56 and 877 for type 2 diabetes, 31 and 348 for tobacco addiction, 31 and 256 for dyslipaemia, 8 and 123 for atrial fibrillation and 17 and 136 for thrombo-embolic risk (totals: 202 and 2392). The clinical cases covered the various clinical situations encountered in the CPGs, and the various events that may be observed in therapeutic histories, such as poor drug tolerance. These tests were used during the development of the ASTI 3 critiquing module; at the end of the development, all tests were passed without error.

Second, we generated decision trees for the diabetes type 2, tobacco addiction, dyslipaemia, atrial fibrillation and thrombo-embolic risk knowledge bases. In these decision trees, each path is a patient profile (including both clinical elements and therapeutic elements, such as past treatment or drug intolerance) and leads to the list of treatments that are not critiqued when prescribed to this patient profile. We have already used such decision trees in a previous study, for type 2 diabetes [19]; an excerpt of a tree is shown in Additional file 3. The decision trees were reviewed by the DSS designers, and they helped to identify some errors in the knowledge bases. For instance, in the dyslipaemia knowledge base, a recommendation was stating that fibrates are less effective than statins; however this is only true when treating hypercholesterolaemia, but not other dyslipaemia such as hypertriglyceridaemia or hypoHDLaemia. We discovered the problem on the tree, and we modified the recommendation by adding hypercholesterolaemia to its condition.

Third, the six knowledge bases were reviewed by a physician. The format of the recommendations expressed in the knowledge bases was clear to the physician. For tobacco addiction, atrial fibrillation and thrombo-embolic risk, the physician found that the knowledge bases conformed to the content of the CPGs. For dyslipaemia, the physician found two errors, related to the use of fenofibrate + statin bitherapy and the definition of the high cardio-vascular risk for diabetic patients; these errors have now been corrected in the knowledge base. For diabetes type 2, the evaluation led to three modifications: a rule has been added for critiquing some sub-optimal bitherapies, and the two rules for insulinotherapies have been modified. For hypertension, the evaluation led to three modifications: quadritherapies and alpha-blocker/central antihypertensives have been allowed under certain circumstances, and diabetic patients with renal failure but without micro-albuminuria were not correctly dealt with. In addition, the physician successfully discovered some recommendations that were present in the CPGs, but that were knowingly not implemented in the knowledge bases, due to practical problems. For example, the tobacco addiction CPG does not recommend hypnosis therapy, however this was not computerized because hypnosis is not coded in the prescriptions of patients' electronic record.

We also measured the system response time for the test base. The system response time was short: about 200 milliseconds for initializing and loading a knowledge base, and then about 35 milliseconds for handling one case (measured on a Pentium 4 processor at 2 GHz with 512 Mb).

## Discussion

In this article, we have highlighted the importance of translating the recommendations found in CPGs into "if conditions then criticism" rules that can be used to criticize physicians' activities during his/her practice; we also describe algorithms to perform the translation automatically from a structured model of CPG recommendations. We propose eight generic recommendations, which are guideline-independent but apply in many situations. DSSs must take into account these generic recommendations, but as they are usually implicit in CPG writing them is not easy. Finally, we describe a method for generating an appropriate textual critique to show to the physician. This task is not trivial because, as CPGs usually do not contain information explaining why a treatment should not be prescribed to a given patient, the existent structured models of CPGs [27] do not represent this information. In this paper, we propose a model of CPG therapeutic recommendations that provides attributes for representing the various elements of the textual critique.

The algorithms we propose can translate all recommendations that fit the CPG recommendation model into "if conditions then criticism" rules, and this model was able to structure all therapeutic recommendations found in the five CPGs. Therefore, it is likely to be pertinent to most or even all situations frequently encountered in general practice. However, further evaluations should be performed to determine if this model can be used as-is for more complex or specific medical fields, such as oncology. In particular, we did not take treatment durations into account; however it would not be difficult to add a duration attribute to the treatment model.

In addition, CPGs also include recommendations for test ordering and diagnosis. Test ordering shares many features with drug prescription, and a method similar to the one we described could be used to criticize test ordering.

Some generic recommendations do not apply to all the five CPGs, and especially the tobacco addiction CPG. This is because tobacco addiction treatment is not a chronic treatment: after the patient has stopped smoking, the treatment can be discontinued. However, despite these exceptions, the generic recommendations we propose sound logical to physicians and we think they can apply in most situations. They can be considered as default behaviour, until the CPG explicitly states the contrary.

The textual critiques generated explain why the treatment proposed by the physician should not be prescribed, provides recommendations for the patients, and gives additional references. They are accompanied by a list of suggestion for treatments. This critique structure seems to cover both the information provided by the guideline and the information expected by the physicians.

Most of the critiquing DSSs published in the literature [9,12,14] are based on a list of manually written "if conditions then criticism" rules. As stated in the introduction, writing these knowledge bases is more complex than structuring the CPG and then automatically generating the "if conditions then criticism" rules, as we propose in this article. J. van der Lei et al. [28] expressed a similar opinion. To facilitate the creation of knowledge bases, they recommended separating medical knowledge, *i.e*. that found in the CPG, from the critiquing knowledge, *i.e*. how to perform a critique using the medical knowledge. In our architecture, the critiquing knowledge corresponds to the algorithms translating the structured model of the CPG recommendations into "if conditions then criticism" rules.

A.M. Albisser et al. [29,31] proposed a critiquing DSS for insulino-dependent diabetes, based on a simulator. This simulator is able to predict how glycated hemoglobin and risk of hypoglycemia evolves when the various doses of insulin and oral antidiabetics are increased or decreased. Such simulators are promising, but this approach is disease-specific, since each disease would require a specific simulator.

P. Groot et al. [32] also proposed a critiquing system based on a CPG model; they used the Asbru format, along with model checking. They also highlighted the difficulty of building human readable critiques, but they only proposed a partial solution to this problem. Another example of critiquing system using recommendations as a knowledge base is the ISABEL system [33], which relies on a set of textbook and native language processing tools for producing reminders related to diagnosis.

Most of the efforts involved in designing the ASTI 3 critiquing module was spent on the design of the engine, including the models and the algorithms. Then, implementing the five CPGs was relatively easy, due to the simple CPG model, close to what is expressed in the guideline, and the generic recommendations, which provide default behaviours for frequent tasks such as dealing with doses. Finally, after the implementation of the diabetes type 2 CPG, we had to update the knowledge base to take into account recent developments in medical knowledge related to the use of glitazones. This update led to the modification of two rules in the knowledge base and has been performed in a few hours work. Most of this time was spent in updating the testing base and then testing the system, to ensure that nothing was broken. Consequently, we think that implementing and updating CPGs in the ASTI 3 critiquing module can be quick and practical. It would be interesting to carry out a more rigorous evaluation of the time required for implementing new CPGs, and for updating an already implemented CPG, and a more detailed assessment of any difficulties encountered.

The ASTI 3 critiquing module could be improved by adding support for the standard CPGs models published in the literature [34], such as Proforma [35], Prodigy [36] or GLIF [37]. This could be achieved by designing an automatical tool to translate these models into the simple CPG recommendation model we have proposed.

Despite its simplicity, our model can represent general therapeutical structures similar to the ones used by the standard CGP models. For example, a plan-based recommendation composed of three plans: diet, monotherapy and bitherapy, with the monotherapy plan including metformin as first-line treatment and AGI as second-line treatment, can be represented in our model with nested "one should prescribe" recommendations. It would lead to the following recommendations: "one should prescribe a diet as first-line treatment, a monotherapy as second-line treatment and a bitherapy as third-line treatment" and "if the proposed treatment is a monotherapy, then one should prescribe metformin as first-line treatment and AGI as second-line treatment".

One of the difficulties that may arise during the design of such an automatic translation tool, is the generation of the textual critics that are displayed to the physicians (i.e. the "criticismLabel" attributes in figure 4). The standard CPG models usually include the text of the CPG, however, as said previously, this text expresses recommendations but not critics, and therefore it may not be appropriate for a critiquing system.

Our intention is now to improve the integration of the ASTI critiquing module in EPR, using more user-friendly dialog boxes and coding support tools, in order to make it usable in real clinical situations. In addition, we plan to integrate the critiquing module with various EPR software, including éO Généraliste but not limited to this particular software.

## Conclusion

We have presented methods, including models and algorithms, for critiquing physicians' prescriptions, using a structured representation of the therapeutic parts of the clinical guidelines. Therefore, writing additional knowledge bases is straightforward, and is even facilitated by the use of generic recommendations, i.e. pre-defined recommendations that apply to almost any guidelines. We have also shown how to generate a textual critique that explains why a non-recommended treatment should not be prescribed. These methods have been successfully applied in the ASTI 3 critiquing module, a decision support system which implements five clinical guidelines related to cardiovascular risks. We are now planning to evaluate ASTI 3, including the critiquing module, in a randomized clinical trial, to determinate the impact of the system on medical practices and patient outcomes.

## Competing interests

The authors declare that they have no competing interests.

## Authors' contributions

J-BL, VE, AV, BS, JB, CS, SD, AB, GS, MF and HF designed the system. CR participates to the evaluation of the system. J-BL and AV drafted the manuscript. All authors read and approved the final manuscript.

## Pre-publication history

The pre-publication history for this paper can be accessed here:

http://www.biomedcentral.com/1472-6947/10/31/prepub

## Supplementary Material

Additional file 1 **Example of ASTI 3 recommendations written in Python**. The first part defines the treatment components: diet and various antidiabetics. Drug are defined by the list of corresponding ATC codes (one or more). The second part is a recommendation for choosing monotherapy. The one_should_prescribe statement starts with the conditions element, specifying that the recommendations applies only when the physician has prescribed a monotherapy *(i.e*. diet + a single oral drug) and the patient's glycosilated haemoglobin is lower than 6.5%. Then the lists of the treatments recommended in first-line (recommended_treatment_linel) and in second-line (recommended_treatment_line2) are given. Each treatment is defined by a list of components, possibly defined at various level of granularity (*e.g*. metformin vs any oral drug); the Any keyword can be used to represent zero, one or more additional components. Treatments and components can also receive additional arguments, for instance "statuts = PRESCRIBED" indicates that the treatment must be the one just prescribed by the physician. Finally, explanation_criticism_labelX and advice_criticism_labelX give the criticism labels for treatment line X (in this example the labels have been shortened compared to the guideline): advice_criticism_labelX is displayed when treatment line X is recommended, and explanation_criticism_labelX when the physician has prescribed a treatment of line X (but not yet recommended). Click here for file

Additional file 2 **The three "if condition then criticism" rules generated by the "one should prescribe" recommendation of additional file **1**, in Python**. Treatments are patterns that are matched with the therapeutic history, and return a boolean value (true if a matching treatment has been found). The first rule corresponds to a patient that has not yet any treatment, and the physician is prescribing a treatment other than AGI and metformin (*e.g*. a sulfamid). It can be read: "If the physician is prescribing a diet associated to an oral antidiabetic, and glycated haemoglobin (A1C) is below or equal to 6.5%, and the prescribed treatment is not diet with metformin, and the prescribed treatment is not diet with AGI, and there is no current or past treatment by diet and metformin that has failed (i.e. not tolerated or inefficient), and there is no current or past treatment by diet and AGI that has failed (i.e. not tolerated or inefficient), then display the criticism". Click here for file

Additional file 3 **Part of a decision tree generated by C4.5, from the knowledge base for dyslipemia. Treatments in brackets are recommended as second-line treatments only**. This excerpt corresponds to the following recommendation in the guideline: "It is not recommended to start a treatment for patients older than 80, in primary prevention", "For dyslipidemic patient in primary prevention, diet should be proposed in monotherapy", "For patient with high cardiovascular risk, drug-based treatment should be started as soon as possible, associated with diet", "Statins are the first-line drug treatment", "Rosuvas-tatin should only be used in case of tolerance or efficacy problems with other statins" and "For diabetic patient, the reduction of the ischemic risk has been demonstrated for atorvastatin and simvastatin". Click here for file
